# Reirradiation of Large or Multifocal High-Grade Gliomas With Gyroscopic Radiosurgery in Combination With Modulated Electro-Hyperthermia

**DOI:** 10.7759/cureus.83511

**Published:** 2025-05-05

**Authors:** Morena Sallabanda, Elisabeth Estefanía Arrojo Álvarez, Borja Aguilar, Ana Lucrecia Ruiz Echevarría, Helena Huertas Modéjar, Kita Sallabanda

**Affiliations:** 1 Radiation Oncology, Institute of Advanced Radiosurgery (IRCA), Madrid, ESP; 2 Oncology, Instituto Médico de Oncología Avanzada (INMOA), Madrid, ESP; 3 Medical Physics, Mercurius Health, Institute of Advanced Radiosurgery (IRCA), Madrid, ESP; 4 Oncology, Instituto Médico de Oncología Avanzada (INMOA), MADRID, ESP; 5 Neurosurgery, Institute of Advanced Radiosurgery (IRCA), Madrid, ESP

**Keywords:** gyroscopic radiosurgery, high-grade glioma, modulated electro-hyperthermia, recurrence, reirradiation

## Abstract

Introduction: High-grade gliomas (HGGs) are the most common primary malignant brain neoplasms in adults, with a high rate of local relapse in the first two years after primary treatment, resulting in poor prognosis. The aim of this study is to describe the potential benefits of the implementation of gyroscopic radiosurgery (GRS) in combination with modulated electro-hyperthermia (mEHT) as a radiosensitizer for the reirradiation of large or multifocal recurrent HGGs.

Methods: A study was designed to evaluate the impact of survival and clinical tolerance. Clinical information of 15 patients treated between April 2023 and September 2024 was analyzed.

Results: Fifteen patients with a median age of 50 years and grade 4 (n = 13) or grade 3 (n = 2) gliomas were included in the study. The median Karnofsky Performance Status (KPS) was 70. Multifocal disease was present in 10 patients. The median time from previous radiation was 16 months. Twelve patients were eligible for analysis. The median planning target volume (PTV) was 33.6 cc; 48% (10 lesions) received five fractions (20-30 Gy), 38% (eight lesions) received one fraction (15-18 Gy), and 14% (three lesions) received three fractions (24 Gy). mEHT was applied every 48 hours. The median follow-up was seven months with no in-field recurrences reported. Actuarial overall survival (OS) from GRS and mEHT was 58.3% at six months and 25% at 12 months. Acute tolerance was acceptable, with 33.3% of patients showing improvement, 33.3% remaining stable, and 33.3% presenting grade 2 radiation necrosis, managed with outpatient steroid adjustment.

Conclusions: High-risk, HGG reirradiation with GRS and mEHT showed a favorable impact on local control and OS with low toxicity. Longer follow-up and larger series are needed to validate these results.

## Introduction

High-grade gliomas (HGG), grade 3 and 4, according to the 2021 World Health Organization (WHO) CNS5 classification system, are the most prevalent malignancies of the central nervous system (CNS) with the most invasive growth pattern. Glioblastoma (GBM), classified as grade 4, represents 54% of all gliomas and 16% of all primary brain tumors. Previously, GBM was diagnosed based on the histologic findings. In WHO CNS5, only IDH wild-type tumors are considered GBM. Furthermore, IDH wildtype diffuse astrocytic tumors in adults with three or more of these gene alterations (EGFR gene amplification, TERT promoter mutation, or combined gain of chromosome 7 and loss of chromosome 10) will also be considered glioblastomas. Moreover, IDH-mutant astrocytomas with the presence of CDKN2A/B homozygous deletion will have a WHO CNS grade of 4. The standard of treatment for HGG is the combination of maximal surgical resection, radiation therapy (60 Gy in 2 Gy per fraction), and concurrent chemotherapy followed by adjuvant temozolomide (TMZ) for six to 12 cycles [1-4].

Despite this multimodal first approach, 40% of WHO grade 3 and 90% of WHO grade 4 are likely to recur within the first two years, and local tumor recurrence within 2 cm of the resection cavity remains unavoidable along the natural history of the disease. On the other hand, distant recurrence (beyond 2 cm of the resection cavity) is rare and commonly occurs in the final stages of the illness. It is typically related to longer progression-free survival (PFS) and positive methylguanine-deoxyribonucleic acid methyltransferase (MGMT) promoter methylation status [1,5].

For patients with recurrence or local progression, the standard of care is not yet defined. Treatment options include repeated surgery, reirradiation, systemic therapy, or a combination. The recommendations are mostly based on retrospective studies, with a high variability of reported PFS, overall survival (OS), and secondary adverse events. Specifically, for patients affected by recurrent GBM, a median OS of less than 12 months is usually reported. The impact of surgery combined with other therapeutic modalities is yet to be defined, but a positive influence in OS is described with an extent of resection > 80%, although increased postoperative morbidity has also been reported, so very specific selection criteria should be defined [1,2,6]. Reirradiation is increasingly used due to technological advances in dose delivery. A higher benefit of this approach has been described in patients with a Karnofsky Performance Status (KPS) > 60, localized/unifocal disease, and an interval with initial radiation ≥ six months. The three most commonly used fractionated regimens include one-to-five-fraction stereotactic radiosurgery (SRS), moderately hypofractionated stereotactic radiotherapy (HFSRT) usually administered in 10 to 15 fractions, and conventionally fractionated external radiotherapy (CFRT). SRS constitutes the most attractive alternative because it offers submillimetric precision, higher conformation, and a steeper dose gradient, reducing overall treatment time and improving acute tolerance. It is reported as the chosen technique in the treatment of lesions < 50-60 cc [5-7]. Regarding systemic therapy, bevacizumab, temozolomide, and lomustine represent the most administered drugs, with no clearly superior regimen identified [8]. In a recently published review and meta-analysis, no significant differences were found regarding PFS and OS between reirradiation and systemic therapy. Nonetheless, combination therapy significantly showed improved PFS and OS compared with monotherapy. Furthermore, the combination with bevacizumab reduced radionecrosis [9].

In the last decades, non-invasive electromagnetic devices have been introduced, showing anti-tumoral effects. These techniques can be used concomitantly and/or palliatively in the treatment of HGG to increase the response to chemoradiotherapy. Modulated electro-hyperthermia (mEHT) and tumor treating fields (TTFs) are non-invasive procedures administered through devices that have to be placed on the skin of the patients. mEHT treatment is recommended three times a week for 60 minutes, while TTF must be worn for >18 hours daily. A recent meta-analysis showed a similar positive impact on GBM survival of mEHT and TTF. TTF has shown benefit mainly in newly diagnosed cases, while mEHT seems to also have a positive impact in recurrent cases [10]. Specifically, mEHT uses a modulated electric field with a carrier frequency of 13.56 MHz generated by two active electrodes to deliver energy to the tumor with the synergy of thermal effects (heat and temperature increase) and nonthermal processes (electron excitations, generating chemical reactions). By these means, a precise, personalized theragnostic selection and treatment of malignancy is achieved, supporting natural homeostatic processes such as apoptosis, immune reactions (antitumoral killer and helper T-cells appear), conditional effects, etc., working as a tumor-specific vaccination [11]. A recent review reveals a growing body of evidence suggesting the potential clinical benefits of electric wave treatments for patients with recurrent HGG [12]. In addition, mEHT has some advantages compared to TTF as more comfort and lower cost, but, as with TTF, it has always been explored as a palliative treatment, which can increase survival but not cure the patient [13].

The aim of this study is to present early results of a series of patients with large unifocal (> 30cc) or any size multifocal recurrence of HGG, which comprises a worse prognosis, treated with gyroscopic radiosurgery (GRS) and mEHT.

## Materials and methods

Study description

This study was conducted at the Institute of Advanced Radiosurgery (IRCA) · Madrid, Spain. A study of reirradiation with GRS in one to five fractions in combination with mEHT for the treatment of recurrent HGG was designed with the purpose of evaluating the survival impact and clinical tolerance. Consequently, the primary objectives of the study are the evaluation of six-month and one-year local control (LC) determined using magnetic resonance (MRI) with gadolinium and OS, together with a detailed description of acute and late toxicity according to Common Terminology Criteria for Adverse Events (CTCAE-v5) [14]. On the other hand, the secondary objectives are the evaluation of intracranial control (tumoral progression outside the re-irradiated areas) and the effects related to the combination with systemic treatment lines. The selected inclusion criteria for this study are age ≥ 18 years, baseline classification on the KPS ≥ 60%, and a time period from previous radiation of at least six months.

The GRS total doses and number of fractions were based on clinical criteria such as tumor size, relationship of the tumor with adjacent critical organs, and time between the previous radiation. Lesion sizes ≤ 5 cc were treated in a single fraction with a total dose of 15 to 18 Gy; lesion sizes > 5 cc and < 16 cc received three fractions with a total dose of 24 Gy; and lesion sizes ≥ 16 cc were treated with five fractions and a total dose of 20 to 30 Gy.

mEHT was applied using an Oncotherm EHY-2000 (Oncotherm, Barcelona, Spain) device every 48 hours (Monday through Friday) during GRS treatment with a 60-minute length using a step-up protocol with a beginning power of 45-60W to a maximum power of 120W covering the whole brain. Patients lie on their side, with a water pillow under the head, and the mobile electrode is located over the head without pressing it. It is administered less than two hours before or after GRS, and after finishing GRS, 2-3 mEHT treatments were administered with the same scheme.

Clinical and physical aspects

Computerized tomography (CT) acquisition without iodinated contrast is required to have a field of view of 50 cm, a matrix size of 512 × 512 voxels, and a slice thickness of 1 mm. It is performed in the supine position, and an athermoplastic mask is used for immobilization. Thin-cut (1 mm) 1.5 Tesla MRI with 3D T1, T2, and T1 five minutes and 60 to 105 minutes postgadolinium are obtained. Rigid and deformable registration is performed between the planning images, and Treatment Response Assessment Maps (TRAMs) are calculated with Brainlab® software (Munich, Germany) to ensure the proper delimitation of the critical structures. TRAMs are obtained by subtracting 3D-T1-MRIs acquired five minutes post contrast injection from those acquired 60-105 minutes post contrast and provide reliable differentiation, with sensitivity and specificity >90% between active tumor cells (colored in blue in the TRAMs) and non-tumor tissues (colored in red), usually compatible with radiation necrosis [15,16].

The gross tumor volume (GTV) is contoured, including the macroscopic areas of contrast enhancement in MRI T1-weighted sequences with gadolinium. TRAMs are used to exclude non-tumor tissues, compatible with radiation necrosis, in order to minimize toxicity. In patients who previously received bevacizumab, pseudo-response and non-enhancement of tumor tissue related to anti-angiogenic effects represent a challenge. In these scenarios, the evaluation of T2/FLAIR abnormalities is important [17]. The clinical target volume (CTV) is considered equal to the GTV in the reirradiation scenarios. The planning target volume (PTV) used is up to 1 mm, according to the treatment delivery precision. Healthy brain (brain-GTV), optic pathway, brainstem, and cochlea are contoured as organs at risk (OARs), and dose restrictions are applied, taking into consideration previous treatments and the number of fractions.

Treatment planning and delivery are performed with ZAP-X®, a new, dedicated, self-contained, and self-shielded GRS system developed and manufactured by ZAP Surgical Systems, Inc. (San Carlos, CA). This 3.0 megavolt (MV) S-band linear accelerator (linac), mounted within a combination of yoked gimbals that accurately rotate around a common isocenter, is intended for stereotactic radiosurgery of intracranial and cervical lesions. Pretreatment patient alignment and intrafraction monitoring are performed with an internally mounted kV imaging system, allowing for a frameless setup and tracking. The ZAP-X linac uses conical collimators ranging from 4 to 25 mm in diameter for beam shaping. In addition, the utilization of very low energy and a very short source-to-axis distance (SAD) of 45 cm optimizes a steep dose gradient. Furthermore, a dose monitor ionization chamber measures real-time radiation delivery to ensure treatment accuracy [18-20].

The ZAP-X GRS platform works with a dedicated treatment planning system (TPS), called ZAP-X TPS. Dose calculation is based on the Ray-Tracing algorithm. A combination of a forward-planning technique to manually place shots in order to achieve an acceptable dose distribution and an inverse-planning optimization using objective functions to obtain an optimal solution regarding beam arrangement is used. Multiple isocenters are often required for conformal dose distribution in irregular target volumes (TVs). The treatment plan is reviewed by a radiation oncologist, a medical physicist, and a neurosurgeon who evaluate isodose distribution, dose-volume histogram (DVH), conformity index (CI), homogeneity index (HI), and gradient index (GI) [21].

Description of the cohort

Fifteen consecutive patients with HGG recurrence were included in a study of GRS in one to five fractions in combination with mEHT between April 2023 and September 2024. The inclusion and exclusion criteria and treatment dose are detailed in the study description. Information about age, gender, date of diagnosis, pathological findings, previous treatments, date and number of recurrences, time between previous irradiation, PTV volume, GRS date, dosimetric characteristics (total dose, number of fractions, coverage, CI, HI, GI, number of beams, and treatment time), acute toxicity at 0 to three months, late toxicity three months after treatment to the last follow-up, and local control status according to periodic two-month follow-up MRIs was collected in a database and analyzed.

Statistical analysis

Frequency tables were created for the descriptive analysis of qualitative variables. Median and range were described in quantitative variables. The date of diagnosis, first recurrence, and GRS-mEHT treatment to the date of appearance of the corresponding event or the date of the last follow-up were analyzed in order to estimate OS using the Kaplan-Meier method. The confidence interval (CI) was designated as 95%.

## Results

Descriptive analysis of the series

Fifteen patients (nine male and six female patients) with a median age of 50 years (range 30-62 years) and histologically confirmed grade 4 IDH-wildtype (n = 13) or grade 3 IDH-mutant (n = 2) HGG were included in a GRS reirradiation with mEHT study. In addition, two patients with grade 4 HGG had MGMT methylation, and one patient had TERT and BRAF mutations. The median KPS before treatment was 70 (range 60-90). Ten patients had multifocal disease, with a median of two lesions treated and a maximum of three. All patients had received the standard Stupp protocol, and five patients had a previous second course of radiation. In addition, 12 patients received at least a second-line systemic therapy (up to four rescue lines), and four patients underwent a rescue surgery before GRS and mEHT. GRS with mEHT was the chosen first rescue treatment in three patients. The median time from previous radiation was 16 months (range six to 60 months).

Twelve patients were eligible for analysis, with at least one two-month follow-up. Diagnostic MRI with TRAMs was performed to determine treatment volume. In grade 3 HGG that presented with extensive low-grade infiltrative components, only areas of high-grade activity observed in TRAMs were treated. The median PTV was 33.6 cc (2.3-63 cc). Eight patients continued with systemic therapies after GRS and mEHT (three patients received bevacizumab in monotherapy, two patients received bevacizumab and lomustine, one patient received lomustine, one patient was treated with temozolomide, and one patient with carboplatin).

Treatment delivery characteristics 

The GRS characteristics are described in detail in Table 1. Treatment was prescribed in one to five fractions, administered on consecutive days in combination with alternating sessions of mEHT. Ten lesions (48%) were treated in five fractions with a median dose of 25 Gy (range 20-30 Gy), eight lesions (38%) were treated in one fraction with a median dose of 15 Gy (range 15-18 Gy), and three lesions (14%) were treated in three fractions (24 Gy). Cumulative equivalent dose in 2 Gy fractions (EQD2) values were lower than 120 Gy. Higher doses were prescribed in out-of-field recurrences.

**Table 1:** Description of gyroscopic radiosurgery treatment delivery parameters

Gyroscopic radiosurgery (GRS) characteristics	Median	Range
Conformity index (CI)	1.19	1.03 - 1.37
Homogeneity index (HI)	1.84	1.22 - 2
Gradiente index (GI)	2.73	2.49 - 3.28
Coverage	95%	89 % – 98.4 %
Isocenters per treatment	8	1 – 16
Beams per treatment	185	90 – 245
Treatment time	40 minutes	37 – 55 minutes

**Figure 1:**
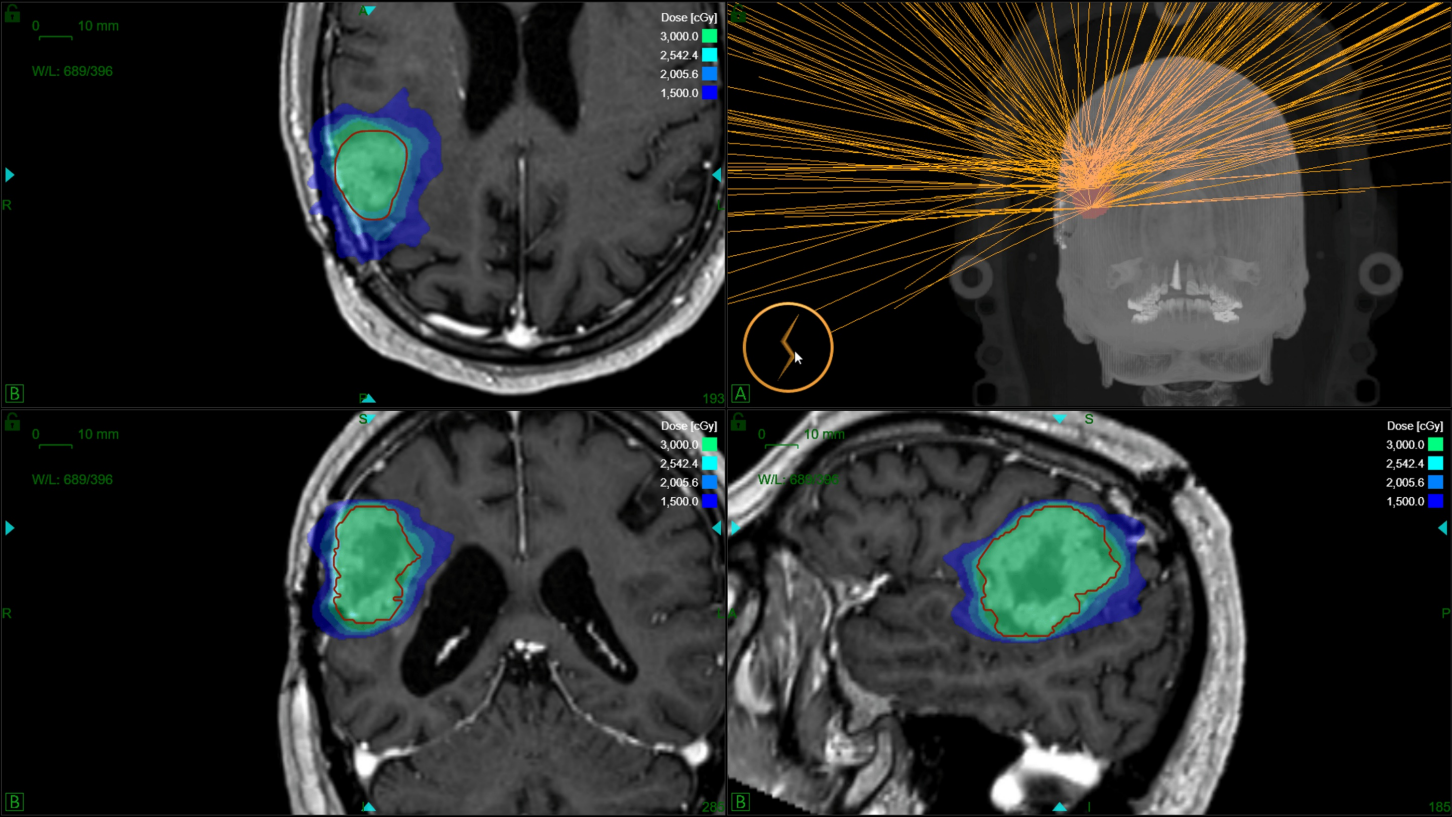
Dose distribution of a gyroscopic radiosurgery reirradiation in five fractions with a total dose of 30 Gy in a patient with recurrent glioblastoma.

All the plans complied with mandatory dose constraints for the optic pathway, brainstem, and healthy brain, prescribed after evaluating previous radiation treatment plans. The basal antiepileptic or corticosteroid regimen was not modified for treatment. In addition, Figure 1 shows the dosimetry of a patient.

Survival and toxicity analysis

**Figure 2:**
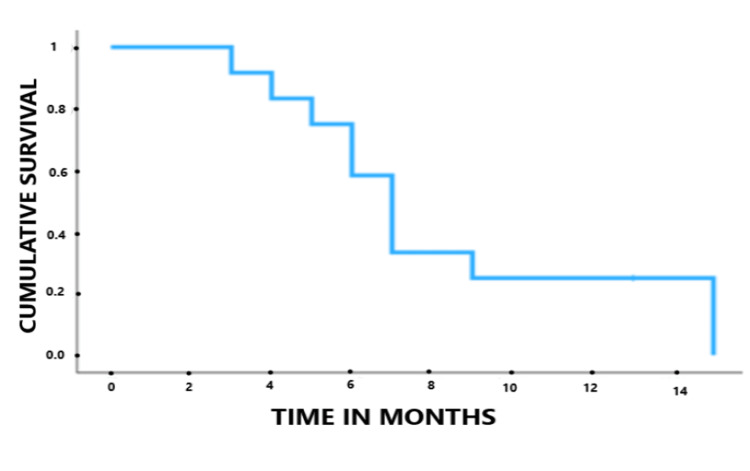
Kaplan-Meier overall survival curve showing an actuarial median overall survival from gyroscopic radiosurgery and modulated electro-hyperthermia of seven months (95% CI 5.9–8.1 months).

The median follow-up from GRS and mEHT was seven months (range three to 15 months). No in-field recurrences have been reported in the follow-up MRIs performed every two months after treatment. Two patients received a second course of GRS and mEHT in new lesions (three and four months after the first GRS). Ten patients died during follow-up. Among them, seven patients had subependymal or multifocal spread (confirmed by MRI), two patients were sedated due to fast clinical progression (without MRI confirmation), and one patient had an infectious complication with respiratory distress. The actuarial median OS from the first recurrence was 22 months. Actuarial OS from GRS and mEHT was 58.3% at six months and 25% at 12 months. The actuarial median OS from GRS and mEHT was seven months (95% CI 5.9-8.1 months). Kaplan-Meier OS from GRS and the mEHT curve were obtained and are shown in Figure 2.

Acute tolerance during the first three-month follow-up was acceptable, with four patients (33.3 %) showing neurological improvement after treatment, four patients (33.3%) remaining neurologically stable, and four patients (33.3%) presenting neurological worsening due to grade 2 radiation necrosis, managed with outpatient steroid adjustment. This radiation necrosis conditioned grade 2 muscle weakness in all these patients, grade 2 depressed level of consciousness in two patients, and grade 2 dysphasia in one patient. Three of these patients with radiation necrosis had two previous courses of radiation, with a higher cumulative EQD2. mEHT didn’t show significant side effects. There were no cases of burnings, epileptic seizures, or bleeding related to the treatment. After the first three months, two patients had a progressive clinical deterioration leading to death, with no further MRI confirmation. The rest of the patients maintained clinical stability until the recurrence of the last follow-up. Clinical tolerance appeared to be more favorable in patients receiving treatment with bevacizumab after GRS and mEHT (four of the five patients showed improvement or stability).

## Discussion

Recurrent HGG management has increasingly been explored in the literature, but the lack of strong prospective evidence and the variability of clinical scenarios have prevented the standardization of a specific approach. Nonetheless, recommendations have been published with the aim of providing basic algorithms to use in daily clinical practice. Life expectancy consideration is the first step to determine eligibility for active treatment. If life expectancy is > three months and the disease is circumscribed, the assessment of the prognostic factors for local treatments and the estimation of expected toxicity should be taken into account, together with the evaluation of the benefits for combined treatments [22, 23].

Regarding expected toxicity in reirradiation scenarios, the estimated risk of radiation necrosis seems to be related to cumulative EQD2, which is used as a risk predictor. Cumulative doses of 120 Gy or lower have been associated with a risk < 10% for a median tumor volume of approximately 10 cc, but a higher risk up to 24% was described for a cumulative EQD2 of > 132 Gy [6].

In addition, the Combs Prognostic Score has been validated as a significant and reliable predictor of survival for patients with recurrent HGG undergoing re-irradiation. Posterior modifications lead to the New Combs Prognostics Score, which sought to include a novel approach, considering the information on resection of recurrent tumors, KPS, and tumor volume, in addition to age, histology, and time between primary radiotherapy. This score classifies patients in four different prognostic groups from “a” (best prognosis with lower scoring value and expected 12-month OS of 88%) to “d” (worse prognosis with higher scoring value and expected 12-month OS of 7%) [24].

The current study investigates the combination of GRS and mEHT as a novel treatment approach for a series of patients with a median classification of scoring group “c” in the New Combs Prognostic Score, with an expected 12-month OS of 22%. This cohort was identified as high-risk recurrent HGG because of large volume or multifocal disease, leading to a worse prognosis. This study is focused on survival outcomes and clinical tolerance. The results of this prospective series highlight several important findings and considerations in the local management of high-risk recurrence in HGG, which will be discussed in the following sections.

Efficacy of GRS and mEHT combination in recurrent HGG

The combination of GRS and mEHT appears to show promising results in terms of local control and OS for patients with recurrent high-risk and multitreated HGG. Notably, no in-field recurrences were reported in the follow-up MRIs, suggesting that the treatment strategy was effective in controlling tumor growth within the treated areas. This outcome is particularly significant given the aggressive nature of HGG and the high risk of local recurrence. The use of GRS allows for precise delivery of radiation, and mEHT contributes synergistically by enhancing the tumor's sensitivity to radiation and promoting immune responses that could further help in controlling the tumor. Though multifocal disease was the main cause of death in these series, the combination with mEHT could have a potential role in slowing up the multifocal relapse.

The data obtained regarding OS from the first recurrence, together with the specific impact of GRS and mEHT, suggest that local treatment combined with a non-invasive technique with anti-tumoral effects does have a role in the final stages of the natural history of this disease and in larger or limited multifocal relapse, when systemic therapy alone is no longer offering adequate tumor control. In addition, most patients could continue receiving systemic drugs after GRS and mEHT.

Toxicity and safety

The treatment was generally well tolerated. Approximately one-third of patients showed neurological improvement post-treatment, and one-third remained neurologically stable. The remaining third experienced neurological deterioration due to radiation necrosis, with acute toxicity being manageable and largely limited to grade 2 effects, which were treated with steroids. This higher toxicity could be related to higher cumulative EQD2. Overall, these findings suggest that the combination of GRS and mEHT does not induce severe adverse effects in most patients, which is consistent with the generally favorable safety profile of these therapies when combined.

Interestingly, patients who received bevacizumab (a VEGF inhibitor) after GRS and mEHT appeared to show more favorable clinical outcomes, with four out of five patients exhibiting stability or improvement. These findings point to the potential benefit of integrating targeted systemic therapies, such as bevacizumab, into the multimodal treatment approach for recurrent GBM, aligning with previous findings that suggest systemic therapy, particularly anti-angiogenic agents, could enhance the effects of local treatments like reirradiation while reducing toxicity [25].

It is important to note that while the treatment's acute toxicity was deemed acceptable, long-term effects will need further evaluation, particularly given the potential for delayed toxicities such as radiation necrosis or cognitive impairments in patients who survive beyond 12 months. The incidence and management of these delayed toxicities will be crucial to determining the long-term viability of this combined treatment approach. Quality-of-life metrics should also be evaluated in the future [26, 27].

Potential advantages of the combination with mEHT

SRS and HFSRT have been widely accepted for the management of recurrent gliomas. In addition, hyperthermia has demonstrated powerful radiosensitizer effects. mEHT is a safe form of hyperthermia that has been shown to effectively sensitize deep tumors, regardless of the thickness of the adipose layers, providing a non-invasive modality that could potentially improve SRS and HFSRT efficacy. Given the effective and selective heating ability to moderate temperatures, the improved tumor perfusion and ability to increase drug absorption, mEHT can be easily applied to sensitize tumors. mEHT also appears to improve local control and survival rates by increasing apoptosis and inducing an abscopal (systemic) response to ionizing radiation. This study's findings on mEHT, however, provide additional evidence supporting the role of electromagnetic therapies in enhancing tumor control without an impact on toxicity [13, 28-30].

Limitations and future directions

Despite the promising early results, several limitations must be acknowledged. First, the sample size is relatively small, and the findings should be interpreted with caution until confirmed in larger, multicenter trials. The lack of a control group further limits the ability to draw definitive conclusions, due to low statistical power, about the relative efficacy of this treatment combination compared to other established therapies.

Additionally, the heterogeneity of the patient cohort, including WHO classification, varying tumor sizes, previous treatments, and performance status, introduces confounding variables that could affect treatment outcomes. The performance of multivariate analysis and stratification by prognostic factors could lead to future studies and should aim to establish more specific selection criteria for optimal candidates for this combined therapy and assess the long-term survival and quality of life outcomes in a larger cohort.

The integration of novel non-invasive therapies like mEHT in combination with GRS should also be explored in more depth, with specific emphasis on the optimal timing, frequency, and duration of treatment. Also, the addition of systemic therapies could impact and alter results compared to mEHT and GRS alone. Further studies should investigate the role of mEHT in conjunction with other emerging treatments, such as immunotherapy or targeted molecular therapies, to understand its full potential in managing recurrent HGG.

Finally, while the use of advanced imaging techniques such as TRAMs for precise treatment planning and monitoring is a strength of the study, it is essential to continue refining these technologies and exploring their role in improving treatment accuracy and minimizing side effects. This is especially important as the patient population grows and as different tumor characteristics are considered.

## Conclusions

In conclusion, the combination of GRS and mEHT shows preliminary promising results in improving local control and OS for a small series of patients with high-risk and worse prognosis recurrent HGG in scenarios where most systemic therapeutic lines have been exhausted. While the treatment appears to be well-tolerated with manageable toxicity, further studies should aim to establish more specific selection criteria for optimal candidates for this combined therapy and assess the long-term survival and quality of life outcomes in a larger cohort. The potential of GRS and mEHT, particularly as part of a multimodal treatment approach, could represent an important step forward in the management of recurrent GBM, offering patients a new option for extending survival and improving quality of life. The role of mEHT, together with other emerging treatments, such as immunotherapy or targeted molecular therapies, is worth exploring in order to understand its full potential in managing recurrent HGG.
